# Successful Switch to Obinutuzumab in a Rituximab-Intolerant Child with Difficult-to-Treat Idiopathic Nephrotic Syndrome

**DOI:** 10.3390/jcm14010239

**Published:** 2025-01-03

**Authors:** Magdalena Drozynska-Duklas, Anna Kranz, Ilona Zagozdzon, Irena Balasz-Chmielewska, Ilona Chudzik, Aleksandra Zurowska

**Affiliations:** Department of Paediatrics, Nephrology and Hypertension, Medical University of Gdansk, 80-210 Gdansk, Poland; anna.kranz@gumed.edu.pl (A.K.); ilona.zagozdzon@gumed.edu.pl (I.Z.); ibalasz@gumed.edu.pl (I.B.-C.); chudzik.ilona@gmail.com (I.C.); aleksandra.zurowska@gumed.edu.pl (A.Z.)

**Keywords:** nephrotic syndrome, children, rituximab, infusion-related reactions, obinutuzumab

## Abstract

**Background**: Idiopathic nephrotic syndrome (INS) is the most common cause of nephrotic syndrome in children. A hallmark of the disease is the rapid remission of proteinuria following a high dose of steroids. Recurrent disease or steroid dependence are common, leading to a high steroid burden and the introduction of steroid sparing therapy. Anti-CD20 antibodies have been increasingly used with excellent results in complicated INS. Nevertheless, their use can be limited by the occurrence of infusion-related reactions (IRRs). **Methods**: This report discusses further treatment options for children who are intolerant to RTX and presents the first report of a successful switch to obinutuzumab (OBI) for a child with difficult-to-treat steroid-dependent nephrotic syndrome (SDNS) and RTX intolerance who was unresponsive to a desensitization protocol. **Results**: A 12-year-old boy with SDNS since the age of 2, was treated with steroids, cyclophosphamide and cyclosporine A (CsA). Because of the prolonged use of calcineurin inhibitors, a course of rituximab (RTX) was planned. Unfortunately, during first infusion, the boy presented with IRR. A desensitization protocol following the first unsuccessful infusion also failed. Facing the risks of long-term cyclosporine therapy, a decision was made to switch to another type of anti-CD20 antibody. Obinutuzumab infusion with a modified premedication scheme was uneventful. **Conclusions**: Switching therapy to obinutuzumab may be considered an option in nephrotic children who are intolerant to RTX when alternative therapies have been exhausted. The addition of montelukast to premedication and employment of desensitization protocols may decrease the risk of infusion-related reactions to anti-CD20 agents.

## 1. Introduction

Idiopathic nephrotic syndrome (INS) is the most common cause of nephrotic syndrome in children. A hallmark of the disease is the rapid remission of proteinuria following a high dose of steroids. Unfortunately, 40% of initially treated subjects will become steroid-dependent (SDNS) or will have a frequently relapsing (FRNS) course of disease [1]. This necessitates the introduction of steroid sparing therapies, which include cyclophosphamide/chlorambucil, mycophenolate mofetil (MMF), calcineurin inhibitors (CNIs) and anti-CD20 antibodies. According to current guidelines, rituximab (RTX) is reserved for difficult-to-treat patients who relapse or develop side effects of previous immunosuppressive (IMS) therapy [2]. RTX is usually well tolerated and has been increasingly used in children with nephrotic syndrome burdened with recurrent relapses and multiple side effects of steroids and immunosuppressive therapy [3]. In a subset of subjects, its administration poses problems due to infusion-related reactions (IRRs), which may be severe or even life threatening [4]. Most of the published data come from adult hematology or rheumatology subjects in whom lymphocyte B depletion therapy is widely used. The biological character of RTX and its murine component implicates a risk of acute reactions such as anaphylactic shock, allergy or excessive cytokine release. The etiology of IRRs is complex and can be immune-mediated (allergic, mainly IgE-mediated) or non-immune-mediated [5]. Mandatory pre-infusion prophylaxis with corticosteroids, antipyretics, antihistamines and a slow rate of infusion has decreased the risk of IRRs. In subjects who demonstrate IRRs despite premedication, desensitization schemes have been used to induce temporary tolerance to RTX [6,7,8]. There are limited data on the safety of switching to other anti-CD20 antibodies in subjects intolerant to RTX [9]. The incidence of IRRs seems to vary depending on the treated disease, with them being more common in oncological than in rheumatological diseases [6,7,10]. There is a paucity of data on severe IRRs in children treated with anti-CD20 therapy for nephrotic syndrome. We present the first report on the successful switch to obinutuzumab (OBI) in a child with nephrotic syndrome intolerant of RTX and unresponsive to a desensitization protocol. We present the different strategies used by oncologists and rheumatologists in subjects with severe IRRs to RTX to raise awareness for nephrologists of the possibilities for the further use of anti-CD20 therapy in their patients.

## 2. Case Presentation

A 12-year boy had been treated for SDNS since he was 2 years old. During 4 years of cyclosporine A (CsA) therapy, he demonstrated relapses requiring the reintroduction of short courses of steroids. At the age of 6 years, he demonstrated a severe relapse complicated by acute kidney injury. This time he required prolonged high-dose steroids to achieve remission. This was followed by the administration of a 12-week course of p.o. cyclophosphamide after which he relapsed 6 months later. A renal biopsy performed at this time revealed a pattern of focal and segmental glomerulosclerosis (FSGS). CsA was again introduced but relapses were frequent. At the age of 9 years he qualified for RTX therapy (375 mg/m^2^ body surface area). Despite premedication, which included methylprednisolone, acetaminophen and cetirizine, he developed symptoms of drug intolerance during the initial minutes of infusion: tachycardia, urticaria covering >10% of body surface area (BSA) and a scratchy throat, followed by cough and dyspnea, which necessitated the early interruption of the infusion. These symptoms were classified as grade 2 according to the CTCAE scale [11]. A standard anaphylaxis therapy, including oxygen, salbutamol, hydrocortisone, 0.9% natirum chloride infusion, and another dose of cetirizine, was introduced with positive result. CsA was reintroduced and he was referred to our center as a difficult-to-treat INS patient, who had been on CsA treatment for 80 months and was running out of therapeutic options. We made a second attempt at administering RTX (375 mg/m^2^ BSA) following a desensitization protocol adjusted for pediatric patients that we use [8]. This involved a three-stage infusion of RTX in increasing concentrations. He tolerated two lower RTX concentration infusions (5.81 mg/250 mL and 58.1 mg/250 mL) well, but during the third infusion (RTX 581 mg in 250 mL 0.9% NaCl), he demonstrated chills and fever, which did not respond to antipyretics, fluids and antihistamines for 48 h of observation. An increase in inflammatory markers (C-reactive protein (CRP)—29 mL/L and procalcitonin (PCT)—10.32 ng/mL) was noted. These symptoms were also classified as grade 2 according to the CTCAE scale [11]. The infusion was stopped and only about 15% of the total planned dose was administered. We could not exclude a cytokine release or allergic reaction and decided against further attempts at administering RTX. Both attempts at biological therapy induced CD20 depletion which lasted for a short period. After the second RTX infusion, CD20 lymphocytes recovered within less than 4 months, which coincided with another nephrotic syndrome relapse. Following reports on the successful use of obinutuzumab in 16/17 hematological adult subjects intolerant to RTX, we decided to rechallenge our nephrotic child with this class II anti-CD20 drug [12]. Permission was obtained from our local Bioethics Committee for the off-label use of OBI at a dose of 1000 mg (18 mg/kg) to treat a child with nephrotic syndrome intolerant to RTX (KB/367/2004). We modified the standard premedication of methylprednisolone, acetaminophen and cetirizine by the addition of montelukast. The drug infusion was performed according to a published desensitization protocol [13]. This involved supplying two bags with two different concentrations which were prescribed stepwise with a slow increase in flow rate. The total dose was delivered over 2 days. A second dose was given 14 days later in the same manner. Following the initial infusion of obinutuzumab, a slight increase in CRP (37 mg/L) and PCT (6.89 ng/mL) was noted without any evident clinical adverse symptoms. The second infusion administered 14 days later was uneventful. The boy has now been in sustained remission for 6 months and is not receiving any concomitant steroids or additional IMS therapy for the first time in 10 years.

## 3. Discussion

RTX infusions are generally well tolerated, in spite of frequent, usually mild, infusion-related reactions in up to 43.8% of subjects [14]. Nevertheless, severe allergic reactions, including life-threatening symptoms, have also been reported [4]. Most of the published data come from adult hematology or rheumatology subjects in whom lymphocyte B depletion therapy is widely used [4,10,14,15,16,17]. The incidences of these reactions seem to vary depending on the treated disease, being more common in B-cell lymphomas than in rheumatological diseases [10]. Due to the frequency of IRRs, premedication (methylprednisolone, antihistaminic drugs and acetaminophen) is mandatory and a stepwise increase in the rate of infusion is used [18]. A subset of subjects still develop IRRs. In oncological patients in whom anti-CD20 therapy is crucial, desensitization schemes have been introduced [8,19].

There is a paucity of data on the incidence of IRRs in children with nephrotic syndrome, though their off-label use in this cohort has been steadily increasing in the last years [20]. Pediatric nephrology publications on RTX administration have focused on its later adverse effects: hypogammaglobulinemia and increased risk of infections. These have guided recommendations for its use in difficult-to-treat steroid-dependent subjects when other agents have failed [2]. This was the case in our subject in whom the treating physician decided to administer anti-CD20 antibodies late, after 80 months of previous IMS therapy. RTX is a chimeric mouse–human anti-CD20 monoclonal antibody directed against CD20 antigen present on B-cells. Its biological character and murine component implicate a risk for acute reactions such as anaphylactic shock, allergy or excessive cytokine release. IRRs have been classified according to their etiology as immune-mediated (allergic, mainly IgE-mediated) or non-immune-mediated reactions, though the two subtypes may be difficult to differentiate [5]. A further role in the development of adverse reactions to RTX has been attributed to the presence of antibodies to rituximab [21]. Mandatory pre-infusion prophylaxis with corticosteroids, antipyretics and antihistamines and a slow rate of infusion decrease the risk of IRRs. In B-cell lymphoma subjects, corticosteroids seem to play a pivotal role in diminishing the risk of infusion-related adverse reactions [22]. This may also be the case in nephrotic subjects who receive RTX after achieving remission with high-dose steroids.

In spite of the above precautions, symptoms may appear during the first infusion, as in the presented child, or following subsequent infusions [4]. Further attempts of drug administration are usually not recommended as they carry a risk of severe life-threatening symptoms. Nevertheless, when alternative therapeutic options are limited, as was the case in our SDNS/FRNS child, RTX rechallenge may be considered, employing strategies that have been shown to successfully diminish the risk of IRRs. The first option is to introduce a desensitization protocol to achieve temporary tolerance to the drug. This should be carried out either in an intensive care setting or with access to rescue medications, because severe allergic reactions may develop during the infusion [6,7,8,23]. There are few publications concerning RTX desensitization and none of them have involved children with nephrotic syndrome [6,7,8]. In hematological and rheumatological subjects, the whole planned RTX dose is divided into three bags which are administered sequentially. The first bag contains a small dose at a very low concentration of RTX. The second bag delivers a 10-fold higher dose at 10-fold higher concentration and the last bag contains the majority of the prescribed dose at a 100 times higher concentration compared to the initial bag. The procedure consists of over a dozen steps, during which the flow rate is increased stepwise [5,6,7,8]. This protocol enabled the uneventful delivery of a small dose of RTX at a low concentration to our subject, but did not prevent symptoms developing when the highest dose was administered, and the infusion had to be interrupted. It is sometimes possible to differentiate the etiology of the IRRs. Skin allergy tests can help to differentiate between allergy and cytokine release symptoms, but were not attempted in the presented case due to the high steroid dose the boy was receiving. Tryptase or serum histamine levels have also been used to differentiate between allergy and cytokine release etiology [5,24], but were not available at the time of symptom presentation in our subject. The repetitive character of the symptoms suggested an allergic reaction and further attempts of RTX administration were halted. A second strategy to be considered for RTX-intolerant subjects is to switch to a different anti-CD20 agent, e.g., ofatumumab or obinutuzumab. Obinutuzumab is a humanized type II anti-CD20 antibody. Because it is glycoengineered, its effectiveness is much higher compared to RTX [25]. Infusion-related reactions during OBI administration have also been reported and also seem to be relatively frequent [8]. Respective desensitization protocols for OBI have also been introduced for hematologic use [8,13]. Some authors have suggested an important role for montelukast, a leukotriene receptor antagonist, in premedication [26].

To date, there are only three publications reporting the off-label use of obinutuzumab in children with nephrotic syndrome and they are focused mainly on its efficacy, though, in the presented series, several patients received the drug due to RTX intolerance [27,28,29]. The two most frequently cited publications come from the same French authors [27,28], who present a successful therapy with OBI in children with complicated nephrotic syndrome. The treatment efficacy they described contributed to reconsidering biological treatment in the presented patient. We decided to switch our patient to obinutuzumab, modifying its premedication by including montelukast and employing a desensitization protocol published for hematological patients [8]. There are scarce data reported in hematologic patients on the difference in the risk of IRRs between RTX and OBI therapy. It has been speculated that their different chemical structures, as well as their different modes of action, may imply different tolerances of infusion [30]. Rituximab induces an average activity of antibody-dependent cell-mediated cytotoxicity (ADCC), complement-mediated cytotoxicity (CDC) and antibody-dependent cellular phagocytosis (ACDP). Obinutuzumab is a very potent inducer of ADCC, ADCP and direct cytotoxicity and only weakly induces CDC (weak complement involvement) [30]. A single study has reported that anti RTX antibodies did not cross-react with obinutuzumab in examined samples from patients treated with other anti-CD20 antibodies [9]. A previous case report from Vivarelli et al. showed that ofatumumab could be used safely in a nephrotic child intolerant to RTX [31]. We can add that it may be possible to switch to obinutuzumab in such a situation, though this probably requires modifying premedication with the addition of montelukast and adherence to a demanding desensitization protocol. Until further observations are reported, and ongoing clinical trials with OBI in children with nephrotic syndrome are finalized, the use of obinutuzumab should be restricted to cases where other therapeutic options have been exhausted. In conclusion, it seems that severe IRRs to RTX do not necessarily limit the use of this efficacious B-cell-depleting therapy for difficult-to-treat nephrotic syndrome in children, due to the possibility of employing alternative anti-CD20 agents.

## 4. Conclusions

Switching therapy to obinutuzumab may be considered an option in nephrotic children intolerant to RTX when alternative therapies have been exhausted.

The addition of montelukast to premedication and the employment of desensitization protocols may decrease the risk of infusion-related reactions to anti-CD20 agents.

## Data Availability

Data are unavailable due to privacy and ethical restrictions.
